# Association between levels of receptor binding domain antibodies of SARS-CoV-2, receipt of booster and risk of breakthrough infections: LA pandemic surveillance cohort study

**DOI:** 10.1038/s41598-023-47261-y

**Published:** 2023-11-25

**Authors:** Neeraj Sood, Chun Nok Lam, Eric Kawaguchi, Olivier Pernet, Andrea Kovacs, Jennifer B. Unger, Howard Hu

**Affiliations:** 1 Sol Price School of Public Policy, University of Southern California, University Park Campus, Verna and Peter Dauterive Hall, 635 Downey Way, Los Angeles, CA 90089 USA; 2 Schaeffer Center for Health Policy & Economics, University of Southern California, Los Angeles, USA; 3 Keck School of Medicine, University of Southern California, Los Angeles, USA

**Keywords:** Infectious diseases, Viral infection

## Abstract

Prevention of COVID-19 with vaccine requires multiple doses and updated boosters to maintain protection; however currently there are no tests that can measure immunity and guide clinical decisions about timing of booster doses. This study examined the association between the risk of COVID-19 breakthrough infections and receptor binding domain (RBD) antibody levels and receipt of booster of COVID-19 vaccines. A community sample of Los Angeles County adults were surveyed between 2021 and 2022 to determine if they had a self-reported breakthrough infection. Predictors included RBD antibody levels, measured by binding antibody responses to the ancestral strain at baseline and self-reported booster shot during the study period. Of the 859 participants, 182 (21%) reported a breakthrough infection. Irrespective of the level of antibodies, the risk of breakthrough infection was similar, ranging from 19 to 23% (*P* = 0.78). The risk of breakthrough infections was lower among participants who had a booster shot (*P* = 0.004). The protective effect of a booster shot did not vary by antibody levels prior to receiving the booster. This study found no association between RBD antibody levels and risk of breakthrough infections, while the receipt of booster was associated with lower risk of breakthrough infections, which was independent of pre-booster antibody levels. Therefore, antibody levels might not be a useful guide for clinical decisions about timing of booster doses.

## Introduction

COVID-19 vaccines have proven effective in reducing the risk of hospitalizations and deaths from COVID-19^1,2^. However, there is evidence that the effectiveness of COVID-19 vaccines wanes over time and thus periodic boosters are recommended^3^. The uptake of boosters is low and the optimal timing of getting boosters is unclear. Past research has shown that antibodies specific to the receptor binding domain (RBD antibodies) of the Spike protein of the SARS-CoV-2 virus are highly correlated with presence of neutralizing antibodies^4,5^. This raises the question whether the level of RBD antibodies can be used as a marker for immunity, which in turn can guide decisions about the optimal timing of boosters or other measures to prevent or treat infection. We conducted surveys and serologic tests in a community sample of vaccinated adults in Los Angeles County to estimate the association between RBD antibody levels and the risk of breakthrough infection and how the association between receiving a booster and risk of breakthrough infections is influenced by RBD antibody levels.

## Methods

### Study procedures

Participants in the study were part of the LA County Pandemic Cohort Study. Details of participant selection and study design are described in an earlier study^6^. Participants completed an online or phone survey and a COVID-19 antibody test from July 9 to July 25, 2021 (baseline) to determine vaccination status and protective behaviors such as mask wearing and avoiding social gatherings. Participants were asked to present their vaccine card during the antibody testing to confirm their self-reported vaccination status. Participants who received at least two doses of the Pfizer or Moderna vaccine and one dose of the Johnson and Johnson vaccine were considered fully vaccinated.

**Figure 1:**
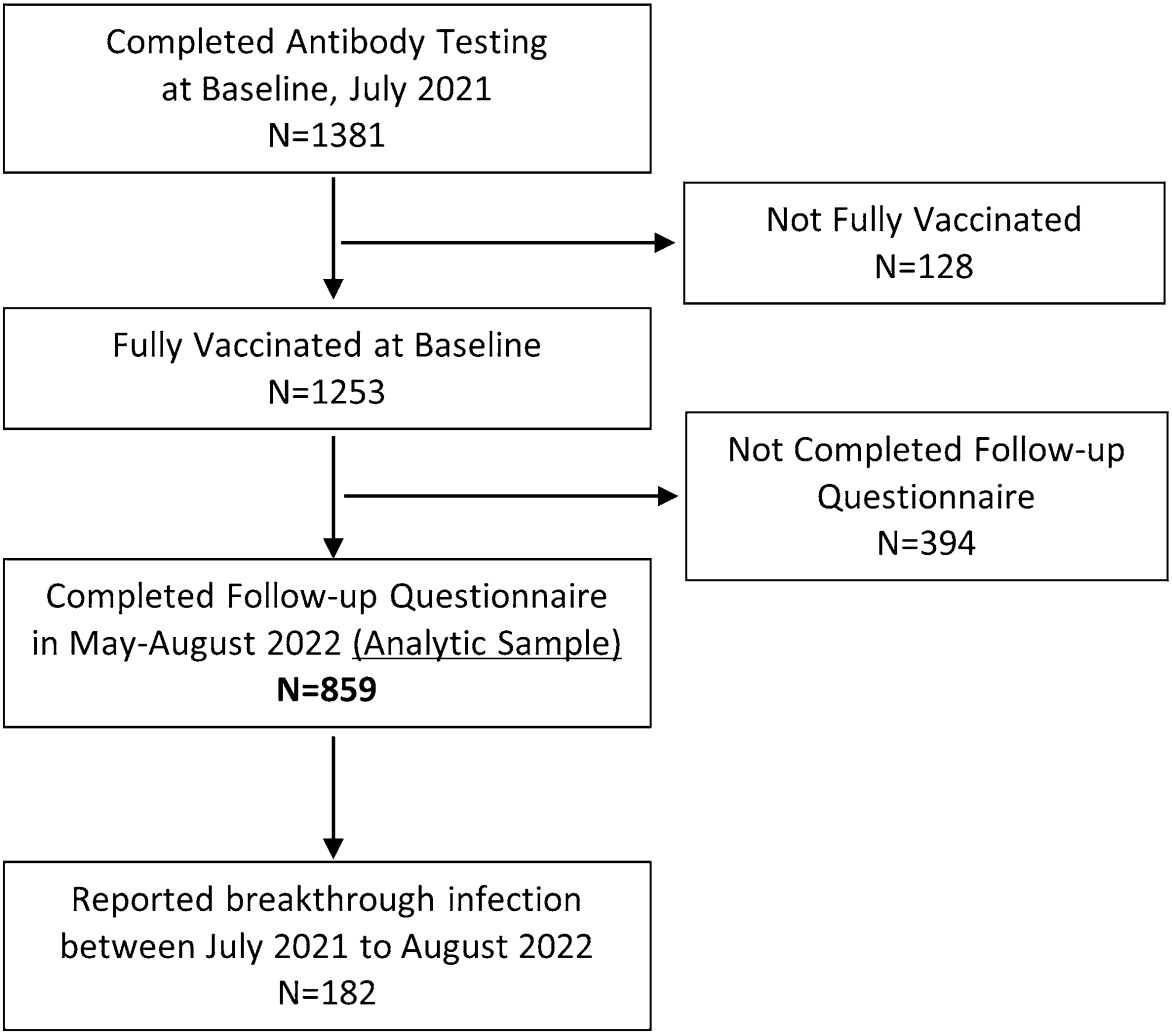
Study CONSORT diagram.

Participants were surveyed again from May 9 to August 16, 2022 (follow-up), to determine if they had a self-reported breakthrough infection reported as a positive COVID-19 PCR or antigen test post vaccination. The study CONSORT diagram is displayed in Fig. 1. Of the 1381 participants who completed the baseline questionnaire and antibody testing, 128 participants were dropped because they reported not being fully vaccinated. Of the 1253 fully vaccinated participants at baseline, 859 participants (69%) completed the follow-up questionnaire, and 394 participants were lost to follow-up. The 859 participants who were fully vaccinated at the baseline survey and completed the follow-up survey were included in the analytic sample for this study. To account for loss to follow-up of roughly 30% of the baseline sample, we used Chi Square tests to compare the characteristics of participants who completed the follow-up questionnaire (analytic sample) and those who were lost to follow-up (Table 1). After conducting the primary analysis using the analytic sample, we re-estimated our main model using weights. We used weights obtained through iterative proportional fitting or raking^7^. The weights were computed so that the distribution of selected demographic characteristics in the weighted analytic sample matched the demographic distribution of the baseline sample (which was recruited to be representative of the population of Los Angeles County). We collapsed some demographic variables with small population sizes for the purpose of raking. We estimated weights to match on the following demographic characteristics: gender (Female, Male or Non-Binary), age (18–29, 20–49, 50+), and race ethnicity (Non-Hispanic White, Hispanic or Black, Asian or Other Race).

**Table 1:** Characteristics of study participants by completion of follow-up questionnaire.

Characteristics	Baseline participants*	Not complete follow-up	Completed follow-up^†^	*P* value
Sample size, no.	1253	394	859	
Gender, no. (%)				
Male	563 (45)	198 (50)	365 (42)	0.02
Female	681 (54)	192 (49)	489 (57)	
Non-binary	9 (1)	4 (1)	5 (1)	
Age group, no. (%)				
18–29	176 (14)	80 (20)	96 (11)	< 0.001
30–49	609 (49)	201 (51)	408 (48)	
50–64	340 (27)	87 (22)	253 (29)	
≥ 65	128 (10)	26 (7)	102 (12)	
Race and ethnicity, no. (%)				
Hispanic	410 (33)	156 (40)	254 (30)	0.001
Non-Hispanic White	465 (37)	120 (30)	345 (40)	
Non-Hispanic Black	81 (6)	23 (6)	58 (7)	
Non-Hispanic Asian	237 (19)	71 (18)	166 (19)	
Non-Hispanic other	60 (5)	24 (6)	36 (4)	
RBD values, baseline, no. (%)				
0–4999	209 (17)	62 (16)	147 (17)	0.58
5000–9999	327 (26)	97 (25)	230 (27)	
10,000–14,999	356 (28)	112 (28)	244 (28)	
≥ 15,000	361 (29)	123 (31)	238 (28)	
Avoided large or small social gathering, no. (%)				
Yes	1036 (83)	318 (81)	718 (84)	0.21
No	217 (17)	76 (19)	141 (16)	
Wore a facemask in the presence of others, no. (%)				
Yes	1111 (89)	350 (89)	761 (89)	0.90
No	142 (11)	44 (11)	98 (11)	

*RBD* receptor binding domain.

*All baseline participants completed antibody testing and were fully vaccinated at the time of participation.

^†^We used this group as our analytic sample.

### Antibody testing

We established 8 testing sites across Los Angeles County. Testing was offered from July 9 to July 25, 2021. We used an FDA Emergency Use Authorized bead-based assay to determine RBD antibody levels according to the manufacturer’s protocol (xMAP® SARS-CoV-2 Multi-Antigen Antibody Assay, Luminex), measuring the binding antibody responses to the ancestral strain. Median Fluorescent Intensity (MFI) values for RBD antibodies were determined. The assay has a dynamic range of 0–25,000 MFI. MFI values ≥ 700 MFI are indicative of SARS-CoV-2 infection or vaccination^8^. The RBD sequence used in our antibody assay is from the ancestral strain, but the assay is not specific to the ancestral strain only. The assay reports all the IgG antibodies targeting any of the RBD epitopes from the ancestral strain coated on the beads of the assay^8^.

### Measurement

The primary outcome was self-reported breakthrough infections. In the follow-up questionnaire, an item asked if the participants ever-had a positive COVID-19 test from a PCR or antigen test, and the date of the most recent positive test. Breakthrough infection was operationalized as a positive test between the baseline and the follow-up questionnaire, as the baseline sample only included individuals who reported being fully vaccinated against SARS-CoV-2. Participants who never tested for COVID-19 or tested negative during the study period were assumed not to have experienced a breakthrough infection. We also estimated time to breakthrough infection as the number of days from the date of antibody testing at baseline to the date of positive COVID-19 test, given our primary interest is in the association between antibody levels and risk of breakthrough infections.

The main predictors tested of breakthrough infections were RBD antibody levels at baseline and self-reported booster shot. The RBD antibody levels were grouped in four categories: 0 to 4999 MFI, 5000 to 9999 MFI, 10,000 to 14,999 MFI, and 15,000 MFI or higher. For the booster shot status, an item in the follow-up questionnaire asked if the participants ever received a booster shot and the date of the booster. Of the 156 participants who reported receiving a booster, 128 had their booster before the breakthrough infection, while 28 had their booster after the breakthrough infection. Given that we included booster shot as a time varying covariate in our analytic model, we treated those who had their booster shot after their breakthrough infection as essentially not having a booster. That is, in our model, receipt of booster only affects the risk of future infections and not the risk of past infections.

Other covariates included demographic characteristics such as gender, age groups, race and ethnicity, as well as self-reported protective behaviors such as mask wearing and avoiding social gatherings. The sensitivity analysis included additional covariates including types of COVID-19 vaccine, days since fully vaccinated at baseline, and prior COVID-19 infections before baseline based on N antibody levels ≥ 700 MFI from the baseline antibody testing.

### Statistical analysis

We used a log-rank test to test the association between the risk of breakthrough infections and antibody levels at baseline. We used a univariate time-dependent Cox proportional hazards model to test the association between the risk of breakthrough infections and receipt of booster shot. Next, we used multivariable time-dependent Cox proportional hazards models to estimate the association between antibody levels, receipt of booster shot and risk of breakthrough infections. In the Cox proportional hazards model time was measured from date of antibody testing, i.e., date of antibody testing was considered day zero. Failure was defined as a breakthrough infection. Observations for participants who did not experience a breakthrough infection were censored at the time of follow-up survey. The key independent variables were antibody levels at baseline and receipt of booster shot, which was modeled as a time-dependent variable. Additional covariates in the model included demographics and the self-reported protective behaviors reported at baseline. In the first Cox proportional hazards model, categorical variables for antibody levels and booster shot were entered as main effects. This model allowed us to examine the association between antibody levels and risk of breakthrough infections as well as the association between receiving a booster shot and risk of breakthrough infections. The second Cox proportional hazards model included the interaction between antibody levels and having a booster shot. This model allowed us to test whether the association between receipt of booster shot and risk of breakthrough infection is moderated by antibody levels.

In sensitivity analysis, we tested the robustness of our main results by controlling for additional covariates including type of COVID-19 vaccine, time since fully vaccinated at baseline, and prior COVID-19 infections.

The study was approved by the Los Angeles County Department of Public Health Institutional Review Board. Written informed consent was obtained from all subjects and/or their legal guardian(s). We confirm that all research was performed in accordance with the relevant guidelines and regulations based on the Declaration of Helsinki. We used Stata Version 15 for the analysis. Two-sided p-values that are less than 0.05 are considered statistically significant. Our report follows the STROBE^9^ reporting guidelines for a prospective cohort study.

## Results

Of the 1381 adults who completed antibody testing at baseline, 1253 (91%) reported being fully vaccinated against SARS-Cov-2 (Fig. 1). Table 1 summarizes the demographic and COVID-relevant behavioral characteristics of the baseline population and follow-up sample (n = 859), of which 489 (57%) were female, 355 (41%) were age 50 and above, and 254 (30%) were Hispanic individuals. Although in comparison to those who were lost to follow-up, the sample that completed follow-up demonstrated some differences with respect to demographics in gender, age and race and ethnicity, they were relatively minor, and the baseline RBD values showed no meaningful differences (*P* = 0.58) (Table 1). We used the follow-up sample as the analytic sample (Table 2), of that 182 (21%) reported having a breakthrough infection with most infections occurring during the winter Omicron BA.1 surge, between December 2021 and February 2022. Supplementary Figure 1 shows the timing of booster infection and breakthrough infection among participants who received a booster shot (n = 156). During our study period between July 2021 and August 2022, the Delta variant (B.1.617.2) was the primary variant circulating Southern California and causing COVID-19 infections in summer and fall 2021. The Omicron variant (B.1.1.529) became the dominant variant in December 2021 and majority of breakthrough infections in our study occurred when Omicron was the dominant variant.

**Table 2:** Proportion of breakthrough infection by RBD values and booster shot (N = 859).

	No breakthrough infection	Had breakthrough infection	*P* value
Sample size, no. (%^†^)	677 (79)	182 (21)	
RBD values, baseline, no. (%^†^)			
0–4999	117 (80)	30 (20)	0.78^**‡**^
5000–9999	186 (81)	44 (19)	
10,000–14,999	188 (77)	56 (23)	
≥ 15,000	186 (78)	52 (22)	
Had booster shot, no. (%^†^)			
Yes	631 (83)	128 (17)	0.004^§^
No	46 (46)	54 (54)	

Sample size of the analytic sample, N = 859.

*RBD* receptor binding domain.

^†^Row percentages.

^‡^*P* value based on log-rank test.

^§^*P* value based on univariate time-dependent Cox proportional hazards model.

**Figure 2:**
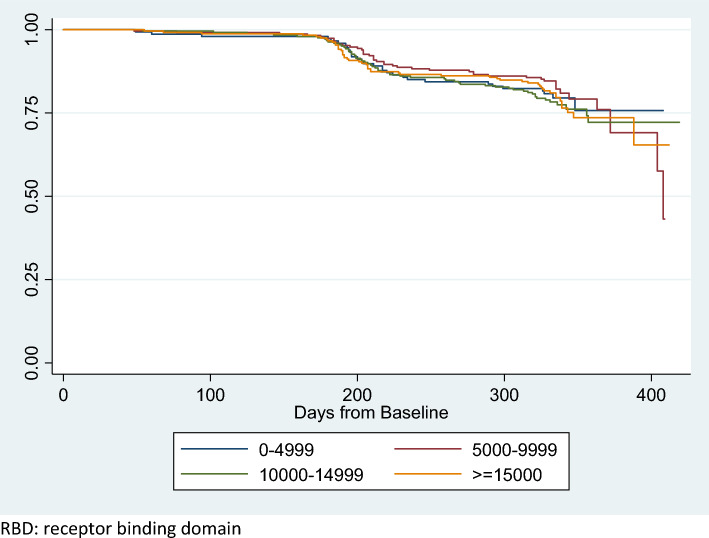
Kaplan–Meier survival estimates by baseline RBD antibody levels. *RBD* receptor binding domain.

Table 2 shows the risk of breakthrough infections by antibody levels and having had a booster shot. Irrespective of the level of antibodies, the risk of breakthrough infection was similar, ranging from 19 to 23%. There was no association between the risk of breakthrough infections and antibody levels (*P* = 0.78). However, the risk of breakthrough infections was lower among participants who had a booster shot. Among those who received a booster shot, 128 (17%) reported having a breakthrough infection, compared to 54 (54%) who did not have a booster shot (*P* < 0.004). Figure 2 shows the unadjusted survival curve by RBD antibody levels.

Table 3 shows the risk of breakthrough infections after controlling for demographic and COVID-relevant behavioral characteristics. Results from the Cox proportional hazards model indicate that baseline RBD values were not associated with the risk of breakthrough infections (*P* = 0.46), while having a booster shot reduced the hazard by 40% (Hazard ratio 0.6, 95% C.I. 0.5, 0.9, *P* = 0.02). Older participants also had a lower risk of breakthrough infections. Supplementary Table 1 tests the interaction between baseline RBD values and having a booster shot; the result shows that the interaction is statistically nonsignificant, suggesting that the association between risk of breakthrough infection and receiving a booster is not influenced by antibody levels at baseline.

**Table 3:** Cox proportional hazards model predicting breakthrough infection.

Characteristics	Hazard ratio	95% CI	*P* value
RBD values, baseline*			
0–4999	Ref		
5000–9999	0.9	0.5, 1.4	0.53
10,000–14,999	0.9	0.6, 1.4	0.65
≥ 15,000	0.7	0.4, 1.1	0.14
Had booster shot^†^			
No	Ref		
Yes	0.6	0.5, 0.9	0.02
Gender			
Female	Ref		
Male/other	1.2	0.9, 1.6	0.33
Age group			
18–29	Ref		
30–49	0.6	0.4, 0.9	0.02
50–64	0.4	0.2, 0.6	< 0.001
≥ 65	0.2	0.1, 0.4	< 0.001
Race and ethnicity			
Hispanic	1.4	0.9, 2.0	0.08
Non-Hispanic White	Ref		
Non-Hispanic Black	1.4	0.8, 2.5	0.27
Non-Hispanic Asian	0.7	0.5, 1.2	0.28
Non-Hispanic other	0.3	0.1, 1.1	0.07
Avoided large or small social gathering			
No	Ref		
Yes	1.0	0.7, 1.4	0.91
Wore a facemask in the presence of others			
No	Ref		
Yes	0.7	0.5, 1.1	0.16

*RBD* receptor binding domain.

*The overall *P* value for RBD values at baseline is 0.46.

^†^Booster is treated as time-dependent covariate.

Supplementary Table 2 includes results from the sensitivity and non-response bias analysis. Model 1 replicates the results from the main model in Table 3, but with re-categorization of gender, age, and racial and ethnic group categories used to create weights to address non-response bias. Model 1 results are similar to the main model, with no association between antibody levels and risk of breakthrough infections and negative association between receipt of booster shot and risk of breakthrough infections. Thus, the results show that the re-categorization of demographic variables does not influence the results. Model 2 shows the sensitivity of results to controlling for additional COVID-relevant variables. The results are robust to controlling for additional variables. Model 3 is a weighted version of Model 2, with weights estimated to match the demographic distribution of the analytic sample to the baseline sample; the results remain unchanged.

## Discussion

We found no association between antibody levels, measured by binding antibody responses to the ancestral strain and risk of breakthrough infections; a finding consistent with a case–control study conducted during the Omicron BA.1–2 dominant wave in Tokyo^10^. However, a prior paper based on testing health care workers in Israel during the time when the Alpha variant was prevalent found that antibody levels were negatively associated with risk of breakthrough infection, suggesting that Omicron variants might have increased immune evasion capabilities^11^. While another paper testing health care workers in Israel during the Omicron wave also found that higher antibody levels were associated with lower risk of breakthrough infections^12^. The study included three shots of vaccine as an inclusion criterion while we use two or one shot based on the vaccine type, and they measured antibody levels 3 to 30 days prior to breakthrough infection. Given our study has a much longer exposure window, it likely explains the difference in findings given waning antibody levels over time.

We found that receipt of a booster shot was associated with lower risk of breakthrough infections, a finding consistent with several other studies^13–15^. However, we also found that the protective effect of booster shots was independent of antibody levels prior to receiving the booster. Taken together these results suggest that antibody levels might be a poor marker of immunity and thus likely to be not useful for guiding clinical decisions about timing of booster doses or other protective measures. This can be explained by several factors such as waning neutralizing antibodies over time, emergence of variants with increased immune evasion abilities and the role of mucosal antibodies such as IgA in protecting against respiratory infections^13–15^. The findings of this study are also consistent with prior work that found that Los Angeles experienced a significant surge in SARS-CoV-2 infections despite a large fraction of Los Angeles population having RBD antibodies^6^. However, it is important to note that the current study did not have a large enough sample to study the association between antibody levels and risk of hospitalization or death. Future studies should confirm this finding by conducting a large prospective study that examines the association between antibody levels and risk of hospitalization and deaths for a representative and diverse sample of participants.

The study has limitations. We used self-reported breakthrough infections. Self-reported infections probably miss asymptomatic infections and those who did not test despite having symptoms. There is no standard assay for measuring binding antibody responses so the results from this study might not be generalizable to antibody levels measured using different assays^8^. Although initially planned, we were unable to repeat antibody testing 3 months after the baseline due to funding restriction. Data regarding change in antibody levels, especially post-booster antibodies, would be an important direction for future studies. While neutralizing antibody may be more predictive of clinical benefit in prevention of COVID-19, our assay focused on the RBD antibodies of the Spike protein of the SARS-CoV-2 virus, which are shown to be highly correlated with presence of neutralizing antibodies^4,5^. Finally, we were unable to estimate the association between binding antibody responses and the risk of COVID-19 related hospitalizations and deaths due to small sample size.

### Supplementary Information

Supplementary Information.

## Data Availability

Chun Nok Lam had full access to all the data in the study and takes responsibility for the integrity of the data and the accuracy of the data analysis. Neeraj Sood and Chun Nok Lam conducted the data analysis. Data are available upon request for replication studies approved by the Los Angeles County Department of Public Health IRB. The datasets generated during and/or analyzed during the current study are not publicly available due to data being collected from a propriety database, but deidentified data specific to the analysis are available from the corresponding author on reasonable request.
