# Supplementary material for: Association between levels of receptor binding domain antibodies of SARS-CoV-2, receipt of booster and risk of breakthrough infections: LA pandemic surveillance cohort study
Source: Sci Rep. 2023 Nov 25;13:20761. doi: 10.1038/s41598-023-47261-y (PMC10676434; doi:10.1038/s41598-023-47261-y)
Supplement: Supplementary file 1 — Supplementary Information. [file 41598_2023_47261_MOESM1_ESM.docx]

**Supplementary Table 1. Cox Proportional Hazards Model Predicting Breakthrough Infection, with Interaction Term between RBD Values and Booster Shot Status**

| **Characteristics** | **Hazard Ratio** | **95% CI** | ***P* Value** |
| --- | --- | --- | --- |
| RBD Values, Baseline |  |  |  |
| 0-4999 | Ref | |  |
| 5000-9999 | 0.7 | 0.3, 1.7 | .40 |
| 10000-14999 | 0.8 | 0.4, 1.8 | .62 |
| ≥15000 | 0.7 | 0.3, 1.6 | .43 |
| Had Booster Shot ^*^ |  |  |  |
| No | Ref | |  |
| Yes | 0.6 | 0.3, 1.3 | .20 |
| RBD Values x Booster ^†^ |  |  |  |
| 5000-9999 x booster | 1.4 | 0.5, 4.1 | .56 |
| 10000-14999 x booster | 1.2 | 0.4, 3.1 | .77 |
| ≥15000 x booster | 0.9 | 0.4, 2.4 | .86 |
| Gender |  |  |  |
| Female | Ref | |  |
| Male / Other | 1.2 | 0.9, 1.6 | .34 |
| Age Group |  |  |  |
| 18-29 | Ref | |  |
| 30-49 | 0.6 | 0.4, 0.9 | .02 |
| 50-64 | 0.4 | 0.2, 0.6 | <.001 |
| ≥65 | 0.2 | 0.1, 0.4 | <.001 |
| Race and Ethnicity |  |  |  |
| Hispanic | 1.4 | 1.0, 2.0 | .08 |
| Non-Hispanic White | Ref | |  |
| Non-Hispanic Black | 1.4 | 0.8, 2.5 | .26 |
| Non-Hispanic Asian | 0.7 | 0.5, 1.2 | .22 |
| Non-Hispanic Other | 0.3 | 0.1, 1.1 | .08 |
| Avoided Large or Small Social Gathering |  |  |  |
| No | Ref | |  |
| Yes | 1.0 | 0.7, 1.4 | .89 |
| Wore a Facemask in the Presence of Others |  |  |  |
| No | Ref | |  |
| Yes | 0.7 | 0.5, 1.1 | .17 |

^*^ Booster is treated as time-dependent covariate

^†^ The overall *P* Value for the interaction term is .84

RBD: receptor binding domain

**Supplementary Table 2: Cox Proportional Hazards Model Predicting Breakthrough Infection with Sensitivity Analyses**

| **Characteristics** | **Model 1** | **Model 2** | **Model 3** |
| --- | --- | --- | --- |
|  | **Hazard Ratio (95% CI)** | **Hazard Ratio (95% CI)** | **Hazard Ratio (95% CI)** |
| RBD Values, Baseline |  |  |  |
| 0-4999 | Ref | Ref | Ref |
| 5000-9999 | 0.9 (0.5, 1.4) | 0.7 (0.5, 1.2) | 0.7 (0.4, 1.2) |
| 10000-14999 | 0.9 (0.6, 1.5) | 0.8 (0.5, 1.3) | 0.8 (0.5, 1.3) |
| ≥15000 | 0.7 (0.4, 1.1) | 0.6 (0.4, 1.1) | 0.6 (0.3, 1.1) |
| Had Booster Shot ^*^ |  |  |  |
| No | Ref | Ref | Ref |
| Yes | 0.6 (0.4, 0.9) | 0.6 (0.4, 0.9) | 0.6 (0.4, 0.9) |
| Gender |  |  |  |
| Female | Ref | Ref | Ref |
| Male / Other | 1.2 (0.9, 1.6) | 1.1 (0.9, 1.5) | 1.1 (0.8, 1.5) |
| Age Group |  |  |  |
| 18-29 | Ref | Ref | Ref |
| 30-49 | 0.6 (0.4, 0.9) | 0.6 (0.4, 0.9) | 0.6 (0.4, 0.9) |
| ≥50 | 0.3 (0.2, 0.5) | 0.3 (0.2, 0.5) | 0.3 (0.2, 0.5) |
| Race and Ethnicity |  |  |  |
| Non-Hispanic White | Ref | Ref | Ref |
| Hispanic / Non-Hispanic Black ^†^ | 1.4 (1.0, 2.0) | 1.4 (1.0, 2.0) | 1.4 (1.0, 1.9) |
| Non-Hispanic Asian / Other | 0.7 (0.4, 1.1) | 0.7 (0.4, 1.1) | 0.7 (0.4, 1.1) |
| Avoided Large or Small Social Gathering |  |  |  |
| No | Ref | Ref | Ref |
| Yes | 1.0 (0.7, 1.5) | 1.0 (0.7, 1.4) | 1.0 (0.7, 1.5) |
| Wore a Facemask in the Presence of Others |  |  |  |
| No | Ref | Ref | Ref |
| Yes | 0.8 (0.5, 1.2) | 0.8 (0.5, 1.2) | 0.8 (0.5, 1.2) |
| Type of COVID-19 Vaccine ^‡^ |  |  |  |
| Pfizer (n=414) |  | Ref | Ref |
| Moderna (n=372) |  | 1.1 (0.8, 1.5) | 1.1 (0.8, 1.5) |
| Johnson & Johnson (n=66) |  | 0.6 (0.3, 1.2) | 0.5 (0.3, 1.0) |
| Not sure (n=5) |  | 2.4 (0.6, 10.1) | 2.2 (0.6, 7.9) |
| Time since Fully Vaccinated ^d^ |  | 1.0 (1.0, 1.0) | 1.0 (1.0, 1.0) |
| Prior COVID-19 Infection |  |  |  |
| No (n=786) |  | Ref | Ref |
| Yes (n=73) |  | 0.7 (0.4, 1.3) | 0.7 (0.4, 1.4) |

^*^ Booster is treated as time-dependent covariate

^†^ The lower CI of Model 1, 2 and 3 are 0.99, 0.97 and 0.96, and being rounded up to 1.0 in the table

^‡^ Two participants with missing data

^§^ Days from fully vaccinated to baseline questionnaire

Model 1: same parameters from Table 3 with minor adjustment on variable categories

Model 2: sensitivity analysis, additionally controlled for three COVID-19 related variables

Model 3: sensitivity analysis, weighted to match demographic distribution of baseline sample

RBD: receptor binding domain

**Supplementary Figure 1. Timing of Booster Vaccination and Breakthrough Infections among Participants who Received a Booster Shot (n=156)**
